# 

**Published:** 1903-05-15

**Authors:** 


### CONTENTS.

Dental Instruction in the Public Schools, *By Prof. Delos Fall* 217

Proceedings of the Seventeenth Semi-annual Meeting of the Southwestern Michigan Dental Society - - 225

Replanting Teeth, - - *By G. S. Hadley, D.D.S.* 228

Porcelain, - - - *By O. N. Lanphear, D.D.S.* 234

Failure of Dental Operations, - *By F. L. Platt, D.D.S.* 238

Pyorrhœa Alveolaris: Its Causes and Treatment, *By Eugene J. Wetzel, D.M.D.* 242

The English Dental Degree, - - - - - - - 245

Non-cohesive Gold Fillings, - - *By G. W. Haskins* 250

The Best Material for Filling Pulp Canals - - - 251

Construction of a Matrix, - - *By G. W. Dittmarr* 253

Preparation of the Mouth for Anesthesia, *By Prof. Friteau* 254

Adrenalin and Cocain, - *By Marcel Lermoyez, M.D.* 255

EDITORIAL, - - - - - - - - - - - 257

ANNOUNCEMENTS, - - - - - - - - - 259

OBITUARY, - - - - - - - - - - - 262

INDENTS, - - - - - - - - - - - 264

